# Effect and implementation experience of intensive adherence counseling in a public HIV care center in Uganda: a mixed-methods study

**DOI:** 10.1186/s12879-021-06862-6

**Published:** 2021-11-19

**Authors:** Zubair Lukyamuzi, Samuel Etajak, Thomas Katairo, David Mukunya, Moses Tetui, Aloysius Ssenyonjo, Rhoda K. Wanyenze

**Affiliations:** 1 Makerere University, Johns Hopkins University Collaboration (MU-JHU), Upper Mulago Hill Road, Kampala, Uganda; 2 Makerere University College of Health Sciences, School of Public Health, Kampala, Uganda; 3 Infectious Diseases Research Collaboration, Kampala, Uganda; 4 Busitema University Faculty of Health Sciences, Mbale, Uganda; 5 Sanyu Africa Research Institute, Mbale, Uganda; 6 School of Pharmacy, Waterloo University, Waterloo, ON Canada; 7 Department of Epidemiology and Global Health, Umeå University, 901 87 Umeå, Sweden

**Keywords:** Intensive adherence counseling, People living with HIV, Unsuppressed viral load, Public HIV care center, Uganda

## Abstract

**Background:**

Intensive adherence counseling (IAC) is an intervention recommended by the World Health Organization to improve anti-retroviral therapy (ART) adherence among people living with HIV on ART with unsuppressed viral load; and in 2016, the intervention was implemented in Uganda. This study evaluated the effect and experiences of providing IAC in an urban HIV care center in Kampala, Uganda.

**Methods:**

This was a sequential explanatory mixed-method study that compared viral load suppression during IAC implementation (intervention) to the period before IAC at Kisenyi Health centre IV. Data were abstracted from patient files and viral load register. The effect of IAC on viral load suppression and associated factors were analyzed using modified Poisson regression with robust standard errors. Using in-depth interviews and an inductive analysis approach in Atlas-ti 8. We also explored experiences of providing IAC among healthcare workers.

**Results:**

A total of 500 records were sampled: 249 (49.8%) in the intervention period and 251 (51.2%) in the pre-intervention period. The mean age was lower during the intervention period 33.1 (± 12.0) than 36.5 (± 13.4) in the pre- intervention period, *p* = 0.002*.* More clients were currently on Protease-based regimen in the pre-intervention period 179 (71.3%) than 135 (54.2%) in the intervention period, *p* ≤ 0.001. In the intervention period, all eligible clients received IAC [249/249 (100.0%)]. Overall, 325 (65.0%) received IAC and of these, 143 (44.1%) achieved viral load suppression compared to 46 (26.3%) who received regular counseling. Receiving IAC significantly increased viral load suppression by 22% (aPR 1.22, 95% CI 1.01–1.47). Clients on Protease-based regimen were less likely to suppress than those on Efavirenz or Nevirapine-based regimens (aPR 0.11, 95% CI 0.08–0.15). All the interviewed healthcare workers lauded IAC for improving ART adherence. However, patient and health care system related factors hindered adherence during IAC.

**Conclusions:**

The full potential of IAC in achieving viral load suppression in this setting has not been reached due to a combination of the patient and health care system related factors. Provision of adequate IAC necessities and use of patient centered approach should be emphasized to obtain the maximum benefit of the intervention**.**

## Introduction

Globally 38 million people were infected with human immunodeficiency virus (HIV) in 2020 [1], despite a 23% decline in new HIV infections since 2010. Overall, 690,000 AIDS-related deaths and 1.7 million new infections occurred in 2019. This contributed to failure to achieve the 2020 targets of reducing AIDS-related deaths to fewer than 500,000 and new HIV infections to fewer than 500,000 [1, 2]. Despite the 38% reduction in new HIV infections in Eastern and southern Africa by the end of 2019, the region remains the most affected [1]. This is partly due to inadequate implementation of available effective strategies and interventions and this will slow the progress towards the ambitious vision of ending HIV/AIDS by 2030 [3]. HIV viral load (VL) suppression is critical in reducing morbidity, mortality, new HIV infections, and drug resistance, and is thus a major strategy in ending HIV/AIDS especially in sub-Saharan Africa (SSA) [4–9]. Various interventions including test and treat strategy [10] have been directed towards expanding access to HIV treatment and achieving a suppressed VL of < 1000 copies/ml of blood. However, unsuppressed VL remains a major challenge in the management of HIV [11–13].

The World Health Organization (WHO) recommends life-long and periodic monitoring of VL to ensure viral suppression and address promptly the common issues for unsuppressed VL [13, 14]. Poor Anti-retroviral therapy (ART) adherence is a major cause of unsuppressed VL, and is responsible for about 75% of detectable VL (VL > 1000 copies/ml) in people living with HIV (PLHIV) on ART [15, 16]. Essentially, the role of ART in HIV management is to suppress VL [10, 13]. Therefore there is an urgent need to maximize ART adherence so that at least 95% of PLHIV on ART are suppressed [11].

To improve ART adherence among PLHIV with unsuppressed VL, the WHO recommends intensive adherence counseling (IAC) [10, 17]. Several studies including systematic reviews, have shown that IAC achieves VL suppression in over 70.5% of PLHIV on ART with unsuppressed VL [18–21]. Moreover, unsuppressed VL among ART treated PLHIV may be due to drug resistance, mal-absorption and drug-drug interactions. However, these may require ART regimen change if they are to be addressed [22]. Therefore, the WHO recommends that if the VL is unsuppressed, IAC intervention should be carried out, followed by a repeat VL test [10]. If the repeat VL is suppressed (< 1000 copies/ml), the client continues with the current ART regimen. Otherwise, virological treatment failure is deduced, and the patient should be switched to another regimen after ensuring that all adherence issues have been addressed [18, 19].

In response to WHO and UNAIDS recommendations, Uganda launched IAC in 2016 [19] in order to achieve the global targets of having 95% PLHIV on ART suppressed [5, 11]. Despite a significant increase in the number of suppressed PLHIV on ART following implementation of such strategies [23], 12% of PLHIV on ART remained unsuppressed in 2018 [2]. Effectiveness of behavioral interventions such as IAC can vary from one context to another [19, 24]. Thus, context-specific evaluations are needed to inform improvements and enhance the effectiveness of such interventions. In the Uganda’s context, health staff positions are 73% filled, there are gaps in provision of laboratory services as well as inadequate integration of HIV health care services in the health system [25]. Moreover, poor quality of counseling, limited skills among health care workers, shortage of health care workforce, lack of patient’s privacy at the facility, patients’ type of regimen, and social demographics may affect adherence intervention outcomes [12, 15, 26–29]. To ensure strategic implementation of IAC and maximize its benefits, there is a need to assess its outcomes following implementation. Therefore, this study aimed to assess the effect and experiences of providing IAC in the first 23 months of implementation in a public urban HIV care center in Uganda.

## Methods

### Study design and population

This was a sequential explanatory mixed-methods study. The quantitative component was a pre-post intervention evaluation. The qualitative component was a phenomenological assessment of health care worker (HCW) experiences. The pre-post evaluation involved reviewing records of all PLHIV on ART who had a VL ≥ 1000 copies per ml on a test done between Jan 2015 and October 2018. Records of patients who were no longer active in the clinic were excluded. The phenomenological assessment involved HCW including clinicians and counselors who were involved in the provision of IAC at Kisenyi Health center IV (KHCIV).

### Study setting

Uganda is located in the East African region with a total population of about 45 million, of which 1.45 million (5.7%) are the PLHIV [2, 30]. The Uganda Ministry of Health (MoH) adopted the WHO IAC recommendations and implemented IAC in various health facilities [31] including KHCIV. KHCIV is the largest urban public HIV care center with catchment population of about 2,000,000 people [32] from the Kampala suburbs, especially the informal settlements. It serves approximately 11,500 PLHIV of whom 1200 were estimated to have a VL > 1000 copies at the end of 2018 [2]. The facility was supervised by Kampala Capital City Authority (KCCA) while the Infectious Disease Institute (IDI) supported the implementation of HIV services. The VL monitoring schedule at KHCIV stipulated performing VL tests for all PLHIV who had received ART for at least six months. Those with VL ≥ 1000 copies/ml received three IAC sessions, one month apart, after which the VL test was repeated. If the repeat VL was suppressed (< 1000 copies per ml), the client continued with the current ART regimen and with a repeat VL test after one year. If VL was unsuppressed (≥ 1000 copies per ml), clients were considered for a switch to a second or third-line regimen after addressing all adherence issues. Testing for possible resistant HIV strains was reserved for suspected failure on a second line regimen before switching to a third regimen [14].

**Fig. 1:**
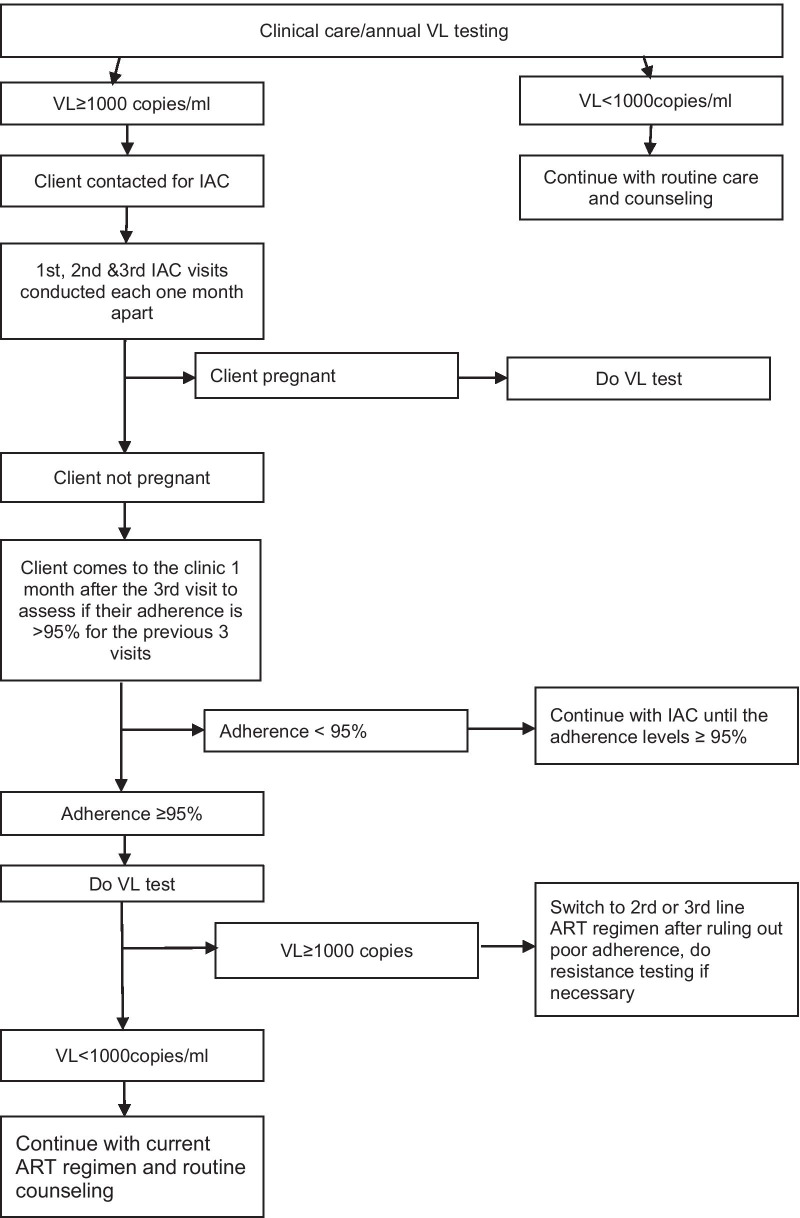
Intensive adherence counseling algorithm at Kisenyi Health center IV

Following confirmation of its effectiveness in earlier studies across the world [21], IDI implemented the IAC intervention in several health facilities across the country, including KHCIV. Implementation of IAC at KCHIV started in early 2016 where some unsuppressed clients on ART received the intervention while others received the regular routine counseling. When the MoH adopted IAC in December 2016, KHCIV made the intervention available to all eligible clients. The HCW (nurses, clinicians and counselors) at the facility initiated the IAC. Expert clients (virally suppressed individuals on ART who volunteered in the ART clinic) also conducted the IAC sessions after orientation and they were supervised by the HCW. For children and adolescents, the sessions were conducted together with their caregivers. Those undergoing IAC were scheduled for monthly sessions for three consecutive months. At these sessions, an effort was made to understand the client’s drug administration, the barriers to adherence, social support, and opportunities to improve adherence using the 5 As (assess, advise, assist, agree, arrange) approach [31, 33]. All the information was recorded in the counseling notes and monitored. At the third session, the client was given a one-month appointment (4th visit). If the adherence was consistently good (≥ 95%) for the previous three visits, the client was prepared for VL testing. Otherwise, a 4th IAC session was conducted. For pregnant clients, VL testing was done at the 3rd visit (Fig. 1).

The Central Public Health Laboratories (CPHL) in Kampala city conducted the VL tests for the entire country. The results were available within two weeks of sample dispatch for delivery to clients at a subsequent clinic visit. If a client was on a second-line regimen and had two detectable VL test results, a resistance test was conducted. All these steps were routinely documented using MOH tools (the VL register, the non-suppressed VL register, and the client files), all of which were source documents for this evaluation.

## Procedures

Data from all relevant records were collected and analyzed. Using 01^st^ December 2016 as the launch of IAC by the MoH, data were divided into two comparable groups. The pre-IAC period included the 23 months before the launch of IAC (01st January 2015–30th November 2016) while the IAC intervention period included 23 months after the launch of IAC (01st December 2016–31st Oct 2018). A pre-tested data abstraction form was used to collect data on the variables of interest from the source documents (VL register, the non-suppressed VL register, and the client files/charts). A list of the ART clinic numbers of eligible participants was extracted from the VL register. Their corresponding clinical charts were retrieved and reviewed for additional information including; age, date of birth, ART start date, and the current ART regimen. Other data included the VL test result and date of the result, whether or not the client had IAC, the dates for the IAC sessions, and the repeat VL test result at the end of IAC. The study outcomes were; (1) participant’s receipt of IAC or not, (2) participant’s achievement of VL suppression following IAC and adherence counseling or not. HCW involved in the provision of IAC were contacted, and their individual interviews were arranged and conducted.

### Sample size and sampling procedures

The sample size was calculated using a formula for two proportions [34], with Zα/2 as the standard normal value corresponding to the level of significance (e.g., for a confidence level of 95%, α is 0.05 and the critical value is 1.96), Zβ as the standard normal value corresponding to 90% confidence interval, β of 0.215 and the critical value of 1.645 and at the power of 80%, the critical value is 0.84. We assumed proportions of VL suppression before IAC (Jan 2015 to Nov 2016) and after (Dec 2016 to Oct 2018) IAC launch at 0.0% and 25.0% respectively [21, 35], and adjusted for incomplete data using 27% [36]. The total sample size was approximately 500 records, i.e., 250 in each group.

Using “2” as the “*K*th” number, $\left( {\frac{{1200\left( {estimated\,unsuppressed\,PLHIV\,at\,the\, end\,of\,2018} \right)}}{{500\left( {sample \,size} \right)}} \approx 2} \right)$, we randomly selected a starting point and thereafter every 2nd record in the generated list of all potential records.

For qualitative sampling, we used the principles of sufficient information power [37] and purposefully selected counselors and clinicians involved in the provision of IAC. Five interviews were conducted in July 2020 (during the phased release of the Covid-19 restrictions) to explore experiences of providing IAC at KHCIV. Interviews were conducted face to face in a private setting. Participants were recruited until the point of saturation [38], when no new themes emerged. The interviews lasted between 15 to 30 min. The first author (Z.L.) conducted all the interviews in English. All the interviews were audiotaped and transcribed by a professional.

### Data management and analysis

Data was entered into excel sheets, cleaned and then transferred to Stata version 14.0 (Stata corp) for analysis. Data were checked for normality and transformation. Frequencies were generated for the number of participants who received interventions (IAC and routine counseling) before and after implementation of IAC, and the number of participants who had VL suppression before and during IAC.

Using “as treated” analysis, frequencies of those who received IAC in either group were obtained. Baseline characteristics were summarized and described into frequencies and percentages for the categorical variables. Descriptive statistics included either mean (± SD) or median with the corresponding interquartile range for continuous variables. Categorical variables were summarized as frequencies and percentages. We determined the factors associated with the VL suppression using the modified Poisson model with robust standard errors to obtain prevalence ratios (PR) and their corresponding 95% confidence intervals. Variables with a p < 0.2 at bivariable level, those known to be associated with VL suppression from literature and those considered plausible although not significant, were entered into a multivariable model to determine the independent factors associated with VL suppression.

The qualitative analysis was an iterative process guided by qualitative content analysis [39, 40]. The analysis included identifying meaning units, abstracting the content of meaning units, and summarizing their importance (Table 1). Words, sentences, or paragraphs that relayed a similar message were grouped as meaning units, condensed, and labeled with a code. We aggregated similar codes to form categories. Categories were made mutually exclusive whenever possible and included all the information related to the content area being discussed. Categories were further analyzed to form manifest sub-themes and themes. We used Atlas.ti 8 (ATLAS.ti Scientific Software Development GmbH, Berlin- Germany). We methodologically triangulated [41] the qualitative and quantitative results at thematic analysis stage, to increase the perspectives and deepen the understanding of the meanings attached to providing IAC at KHCIV.

**Table 1:** Examples of meaning units, codes, categories and themes from qualitative content analysis of interviews about experiences of providing IAC at KHCIV

Meaning units/quotes	Codes	Category	Theme
“We have to be very sure that the adherence is above 95% or the adherence is good so we continue our adherence counseling, the reason why I told you we give an allowance of three to six months to conduct IAC. For adults, we do the 3 sessions, one month apart. Then at the third session if the adherence is good, we give them a one-month appointment to come back for a repeat VL test”	Ensuring good ART adherence	IAC is done to ensure good adherence	IAC is the recommended strategy for PLHIV with unsuppressed VL
“then we are having this category of men and women having disclosure issues, maybe a son or daughter fears to tell the father or mother and they keep dodging around; today he or she takes [drugs], tomorrow he/she doesn’t take; so generally, adherence has those dynamics”	Non-disclosure hinders ART adherence	Factors that hinder ART adherence during IAC	Patient related factors affecting ART adherence during IAC
“At the facility level I can say long time waiting for example; some clients come knowing he/she is going to leave early but takes a whole day at the facility”	Long waiting time at the facility	Issues at the facility affecting client adherence to ART and clinic appointments	Health care system related factors affecting ART adherence during IAC
“So, if we engage these peers, give them the training and mentorship, they can do the counseling very well; so that is what is missing here.”	Peer Educators administering IAC	Reducing the workload of HCW	Facilitating provision of IAC

### Ethical consideration

The study was approved by Makerere University School of Public Health Higher Degrees, Research and Ethics Committee (HDREC). Permission to conduct the study was sought from the KCCA administration. Quantitative analysis was based on routine de-identified data. Informed consent was obtained from the participants in the qualitative interviews.

## Results

### Proportions recruited and analyzed

**Fig. 2:**
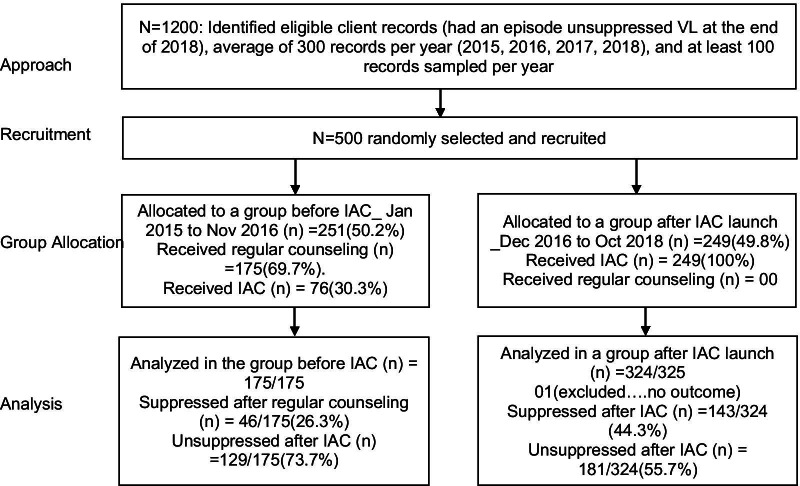
Numbers and proportions of participants at each stage of the study

Overall, 325 (65.0%) received IAC with 249 (76.6%) in the intervention period and 76 (23.4%) in the pre-intervention period. In contrast, 175 (70.0%) of the participants in the pre-intervention period received regular routine counseling and none (0.0%) received regular counseling in the intervention period as summarized in Fig. 2.

### Participant characteristics

Data from 500 client records were analyzed. The mean age was significantly lower in the post IAC introduction group at 33.1 (± 12.0) than 36.5 (± 13.4) in the pre- IAC introduction group, *p* = 0.002*.* More clients in the post IAC introduction group had started ART with Efavirenz-based regimen 187 (75.1%) than 130 (51.8%) in the pre-IAC introduction and this was statistically significant, *p* ≤ 0.001. Less clients in the post IAC introduction group were currently on Protease-based regimen 135 (54.2%) than 179 (71.3%) in the pre-IAC group and this was statistically significant, *p* ≤ 0.001. There were also statistically significant differences in the median CD4 count, first median VL level in HIV care, number of times a client switched a regimen, first median detectable VL level in HIV care and having a CD4 level above or below 500 cells/µl across study groups. However, there were no statistical differences in sex and marital status and the median numbers of days on ART across groups as shown in Table 2.

**Table 2:** Descriptive characteristic of participants stratified by study group

Variable	Pre IAC-introduction (n = 251)	Post IA- introduction (n = 249	P-value
Age in years, mean (SD)	36.5 (13.4)	33.1 (12.0)	0.0028
Sex			0.619
Male	90 (35.7)	84 (33.7)	
Female	161 (64.1)	165 (66.3)	
Marital status			0.962
Married	129 (51.4)	131 (52.6)	
Single	101 (40.2)	98 (39.4)	
Children	21 (8.4)	20 (8.0)	
CD4, median (IQR)	180 (85–310)	261.5 (118–452)	< 0.001
CD4			0.006
500 and less	191 (89.7)	155 (79.9)	
> 500	22 (10.3)	39 (20.1)	
First viral load, median (IQR)	13,551 (2851–47,095)	2298.5 (0–26,508)	< 0.001
Initial regimen			< 0.001
Efavirenz-based regimen	130 (51.8)	187 (75.1)	
Non-Efavirenz-based regimen	121 (48.2)	62 (24.9)	
Current regimen			< 0.001
NNRTI	42 (16.7)	68 (27.3)	
Protease Inhibitors	179 (71.3)	135 (54.2)	
DTG-based regimen	30 (12.0)	46 (18.5)	
Regimen switching			< 0.001
0	73 (29.1)	124 (49.8)	
1	174 (69.3)	124 (49.8)	
2	4 (1.6)	1 (0.4)	
First detectable viral load, median (IQR)	20,782 (20,669–20,847)	21,206 (21,017–21,390)	< 0.001
Days on ART, median (IQR)	878.5 (395–1471)	988.5 (336–1595)	0.648

### Intensive adherence counseling coverage

After introduction of IAC (Dec 2016), all clients with unsuppressed VL received IAC, 249(100.0%) compared to 76 (30.3%) clients in the pre-IAC period. Among those who received IAC, the median number of sessions received was 4 (IQR 4–5), and the median number of days to the first IAC session was 50.5 (IQR 23–84). The median number of days during IAC was 102 (85–142), Table 3.

**Table 3:** Coverage and receipt of IAC

Characteristic	Frequency (N)	Percentage (%)
Cohorts		
Pre-IAC introduction	251	50.2
Post-IAC introduction	249	49.8
Intervention		
Regular counseling (standard of Care)	175	35.0
Intensive Adherence Counseling	325	65.0
Coverage of IAC after Dec 2016(%)	**249 (249)**	**100.0**
Number of IAC sessions, median (IQR)	**4 (4**–**5)**	
Days to first IAC, median (IQR)	**50.5 (23**–**84)**	

### VL suppression and factors associated

On bivariate analysis, 499 participants had VL results following adherence counseling in both groups. Participants in the group after introduction of IAC were 68% more likely to achieve VL suppression (PR = 1.68, *p-*value > 0.001) than those before. Female clients were 12% more likely to achieve VL suppression (PR = 1.12, *p*-value = 0.349) than the males, however, this was not statistically significant. Those who were single were 22% less likely to achieve VL suppression (PR = 0.78, *p*-value = 0.042) than the married ones. Those who had a CD4+ count above 500 cells/µl were more likely to suppress (PR = 1.37. *p*-value = 0.04) than those with a CD4+ count below 500 cells/µl. Clients initially on Efavirenz-based regimen were 36% more likely to suppress (PR = 0.64, *p*-value = 0.001) than those on non-Efavirenz-based regimen. Clients currently on protease inhibitor (PI)-based regimen were 90% less likely to achieve VL suppression (PR = 0.1, *p*-value = 0.001) than those on Efavirenz or Nevirapine-based regimen.

In the final adjusted model, clients in the group after IAC introduction were 22% more likely to achieve VL suppression (aPR 1.22, 95% CI 1.01–1.47) than those before IAC. Clients who were currently on PI-based regimen were 89% less likely to achieve VL suppression (aPR 0.11, 95% CI 0.08–0.15) than those on Efavirenz or Nevirapine-based regimen. Crude and Adjusted Risk Ratios are shown in Table 4.

**Table 4:** Viral suppression following unsuppressed Viral Load from Jan 2015 to Oct 2018 and associated factors

Variable	Not suppressed (n = 310)	Suppressed (n = 189)	Crude PR	p-value	adj PR	95%CI	p-value
Intervention							
SOC	129 (73.7)	46 (26.3)	1		1		
IAC	181 (58.9)	143 (44.1)	1.68	> 0.001	1.22	1.01–1.47	0.04
Age in years	34.6 (13.1)	35.1 (12.5)	1.01	0.666	1	0.99–1.01	0.949
Sex							
Male	113 (36.5)	61 (32.3)	1		1		
Female	197 (63.6)	128 (67.7)	1.12	0.349	1.06	0.08–1.28	0.559
Marital status							
Married	150 (48.4)	110 (58.2)	1				
Single	133 (42.9)	65 (34.4)	0.78	0.042			
Children	27 (8.7)	14 (7.4)	0.81	0.349			
CD4 count							
≤ 500	226 (87.6)	120 (80.4)	1				
> 500	32 (12.4)	29 (19.5)	1.37	0.04			
First viral load	43,982 (35,011.1)	34,107.7 (18,072.5)	0.99	0.496			
Initial regimen							
Efavirenz-based regimen	178 (57.4)	138 (73.0)	1				
Non-Efavirenz-based regimen	132 (42.6)	51 (27.0)	0.64	0.001			
Current regimen							
NNRTI	13 (4.2)	97 (51.3)	1		1		
Protease inhibitors (PI)	285 (91.9)	29 (15.3)	0.1	< 0.001	0.11	0.08–0.15	< 0.001
DTG-based regimen	12 (3.9)	63 (33.3)	0.95	0.429	0.96	0.85–1.09	0.543
First detectable viral load	70,203.2 (199,420.7)	90,402.5 (289,995.8)	1	0.32			
Days on ART	1131.3 (1034.2)	1053.0 (1005.2)	0.99	0.411			

### The Health Care Worker experiences of providing intensive adherence counseling at Kisenyi Health Center IV

In exploring experiences of providing IAC among HCW involved grouping of words, sentences and paragraphs of similar message to form meaningful units. Similar meaningful units were condensed to form codes; similar codes were aggregated to form categories which were eventually grouped to form themes. The major themes included; IAC is an effective intervention recommended for PLHIV on ART with unsuppressed VL, patient related factors affect ART adherence, health care system related factors affect adherence and facilitators of providing IAC.

### IAC is an effective intervention recommended for PLHIV on ART with unsuppressed viral load

The findings from health care workers’ experiences indicate that IAC was viewed as an effective intervention that was fully embraced at KHCIV. Respondents noted that it was a very useful strategy in ensuring ART adherence and achieving VL suppression.

“We have to be very sure that the adherence is above 95% or the adherence is good so we continue our adherence counseling, the reason why I told you we give an allowance of three to six months to conduct IAC. For adults, we do the 3 sessions, one month apart. Then at the third session if the adherence is good, we give them a one-month appointment to come back for a repeat VL test.”

[said a Clinician with 7 years of experience]

Health care workers exhibited a clear understanding of the IAC program and the target group of clients with unsuppressed VL (VL > 1000 copies). A client is considered suppressed with IAC when the VL is less than 1000 copies/ml after repeat VL test. They conducted 3 main IAC sessions and a fourth session in case adherence was not above 95%. In the event of persistently poor adherence, even after the fourth session, the client was referred to the clinician to provide a way forward. On rare occasions the entire IAC process was repeated for some clients.

“Actually, the third session will be the determinant to go to the fourth one. After the 3rd session if you see that this person has really scored 95%, you can stop on the 3rd one and the fourth visit is just for review and then you forward for VL testing. But if you see that he/she is still in 80 or 70% adherence, then you do the fourth session.”

[Said a Counselor with 5 years of experience]

### Patient and health care system related factors

Health care workers said that about 20–30% may fail to achieve VL suppression following IAC. This can majorly be due to several factors which can be both patient and health care related.

#### Patient related factors

These majorly included non-disclosure and socio-economic constraints.

*Non-disclosure* The major patient factor highlighted was non-disclosure. Respondents said that most clients, when they learn about their HIV status either delay or fail to disclose their status to important other people. Before they disclose their status, they take their ARVs in hiding or they may completely fail to take the drugs.

“There are situations that come in for a client to tell you that I am not ready for ARVs for example non-disclosure; this is something that comes to them maybe like a shocker and so they start imagining you are telling them they are going to take ARVs for life, how am I going to go to my partner, how am I going to keep this medicine. Yes, you can give them the medicine because the guidelines say they should take the medicine. It should be noted that for those who fail to disclose, it also gets difficult for them to explain why they would opt for safe sex; this keeps exposing them”

[said a Clinician with 7 years of experience]

“…then we have this category of men and women having disclosure issues, maybe a son or daughter fears to tell the father or mother and they keep dodging around; today he or she takes [drugs], tomorrow he/she doesn’t take; so generally, adherence has those dynamics.”

[said a Counselor with 5 years of experience]

*Social-economic life of the client* This is key to adherence. Patients report challenges like lack of food and say they cannot take drugs on empty stomach. Some clients do not have jobs and hence cannot afford a living.

“…a mother is going to tell you am not working, we do not have food so I cannot take medicine on an empty stomach; so, if the mother is not taking medicine automatically the child will not take medicine if they are also infected and if their issue is food, that is something we might not solve at the facility.”

[said a Counselor with 10 years of experience]

“Yes, because they have their other issues. You can do the counseling very well and they go back home but some have challenges of food; they say they cannot take this drug when they have not eaten anything.”

[said a Counselor with 4 years of experience]

It had been very difficult for the HCW to address the socio-economic constraints. They had linked a few clients especially vulnerable children to some projects for additional support but these institutions cannot take on all the clients due to resource constraints.

“We have the Orphans and vulnerable children (OVC) program but it only looks after children yet there are also adults who are badly off.”

[said a Counselor with 4 years of experience].

Other support encouraged by health workers was psychosocial support from family members or relatives but this was difficult especially for those who have not disclosed. Additional support is also sought from some organizations.

“We are working with some community-based organizations (CBOs) but of course they also make things very long, some say we are full”

[said a counselor with 4 years of experience].

*Other patient related barriers* Some clients initially achieve VL suppression but return with poor adherence due to emerging barriers. These include; relocation with travel related challenges, lost to follow up, peer pressure, getting new partners and gender-based violence (GBV).

“…you are kind of like, you used to do well, what could be the problem! It could be behavior change, it could be nature of work, it could be travelling, it could be distance, and it could be some other things like GBV, so surely it depends. So, a client is like health worker I used to have a good job, so I used to be well.”

[said a Counselor with 10 years of experience].

However, the biggest barrier to VL suppression during the period when these interviews were conducted was the COVID-19 pandemic and related restrictions. Several clients reported missed clinic appointments and drug refills due to transport challenges.

“…because people were in lock down, for about two months some were not taking drugs and they were saying that no car was allowed to move and there were no nearby health facilities for some clients to get drug refills. Many will tell you the lock down got me deep in the village and there is no nearby facility so I was not taking and currently we have got some mothers who are giving birth to positive children because they were not taking drugs during the lock down.”

[said a Counselor with 4 years of experience].

#### Health care system related factors

Health care workers mentioned several healthcare-system related factors that affect adherence during IAC, including provider counseling skills, heavy workload due to big client load, challenges with ensuring privacy, the lack of multi-disciplinary teams to address varied client challenges, and language barriers.

*Counseling skills* It was reported that building rapport with the client is key in solving their ART adherence barriers. If a counselor fails to have a good relationship with the client, then it becomes very difficult for the client to follow their instructions.

“…you know with counseling you need to be closer to the patient as much as possible but as you know government health care workers, for some, that is not part of their job; their job is to come, quickly see patients, give them drugs, and then go. So, when it comes to ideal counseling there is a gap there.”

[said a Counselor with 10 years of experience].

*Heavy client load* The patient-health worker ratio is so high resulting in a HCW attending to many clients in a day. This causes HCW fatigue and increases the client waiting time.

“At the facility level I can say long waiting time for example; some clients come knowing he/she is going to leave early but takes a whole day at the facility.

Another thing I say is manpower; the staff to patient ratio is really not matching, you are a team of twenty and you are looking at a thousand of patients.”

[said a Counselor with 5 years of experience].

Fatigue among health care workers affects the quality of services and this affects adherence due to limited counseling time.

“Of course, it is important. The very first encounter with a HCW if you find this HCW very tired, their attitude may not be good.”

[said a Clinician with 7 years of experience].

*Lack of privacy* Due to many clients, the facility environment may not ensure client’s confidentiality. Clients may thus fear to open up in presence of other clients. This especially happens during group counseling or when the HCW share a clinical room.

“You know sometimes we share offices like in my room I have a colleague I sit with; the counselors also share offices; when the clients are many, they decide to give a group counseling session forgetting that people have got individual issues. So, in that group counseling a client may not open up.”

[said a Clinician with 7 years of experience].

“here we have very big numbers so some clients have stigma; so, when they come here and realize that the numbers are too big, they fear to be seen and thereafter start defaulting their appointments.”

[said a Counselor with 10 years of experience].

*Lack of a multidisciplinary team* IAC requires a multidisciplinary team involving various cadres right from peer educators, counselors, clinicians, pharmacists, psychologists and family members. However, this has not been possible and in most cases only one person or two are available to handle the client’s adherence barriers.

“Okay, ideally intensive adherence counseling involves the multidisciplinary team but I have told you that it is hard to collect that team together; in our setting here, the team is supposed to involve a clinician, a counselor, a pharmacist, an expert client, a family member and a support group. But bringing these persons together to talk to this client is something hard and some are not available.” This means that the workload would be much and one might not be trained enough to do the work of the other. This leaves the patient with scanty information.

[said a Clinician with 7 years of experience].

#### Other barriers

Other barriers include drug side effects and language barrier. It was reported that a few clients may fail to contain the side effects of the drugs and opt for drug holidays. Additionally, the location of KHCIV has many refugees who only speak their own languages and this affects the communication with HCW. The other health care system related barriers included drug resistance, drug stock-out, and long results turn-around time.

### Facilitators of IAC provision at KHCIV

Health care workers mentioned several strategies that had eased their work during provision of IAC including support from the peer educators and assigning special clinic days to clients with adherence challenges.

*Use of peer educators* Peer educators provided support to other clients including support for IAC. This reduced the workload of HCW.

“You know us HCW we tend to think that these peers or expert clients will not give the right information but if these people are empowered, they give that starter information before the client proceed to the counselor because this is a person who has been in the same situation before so they will educate this client basing on what they have gone through.”

[said a Clinician with 7 years of experience].

However, there were some gaps in the peer educators’ strategy at KHCIV such as lack of adequate training and mentorship.

“So, if we engage these peers, give them the training and mentorship, they can do the counseling very well; so that is what is missing here.”

[said a Counselor with 10 years of experience].

*Assigning special clinic days and sessions to special groups or clients* In order to reduce the workload and provide more time to clients, clients with special needs and challenges such as adherence were allocated to special clinic days. These clinics have fewer clients and thus allow more time to support clients with adherence difficulties. At KHCIV, special clinic day and session strategy was introduced and clinics for clients with unsuppressed VL were on Tuesdays.

“Tuesday is for those with unsuppressed VL. So, when these patients come in we let them have a group session with expert client first; then a clinician chip later and there after they are sent to the counselor. Pregnant and breastfeeding clients also have their own sessions; clients also have their family support sessions where they are given some information before they go to the counselor.”

[Said a Clinician with 7 years of experience]

## Discussion

This study aimed to assess the effectiveness of the IAC strategy and staff experiences with its implementation. The study showed that IAC had been embraced in a public HIV care center and there was a clear understanding of the program among HCW. IAC was associated with a 44.1% increase in VL suppression although it did not produce the expected 70% VL suppression. Being on PI-based regimen as the current regimen reduced the likelihood of VL suppression. Non-disclosure, social economic constraints, relocations, gender-based violence, lack of privacy at the facility, limited HCW skills, the Covid-19 pandemic and lack of a multidisciplinary team during counseling hindered VL suppression during IAC. Use of peer educators and special clinic days for clients with unsuppressed VL facilitated provision of IAC at KHCV*.*

The IAC coverage was universal for all eligible sampled clients after the launch of the intervention probably due to an adequate understanding of IAC procedures among HCW. The public facility was supported by IDI, a private not-for-profit organization with extensive experience in HIV care and treatment, which may have influenced its quick rollout and good outcomes. On the contrary, Nassuna et al. found a lower coverage of 68% in various facilities supported by IDI in Uganda [42]. This could have been attributed to different study periods because Nasuuna et al.’s study was done from Jan 2015 to Dec 2016 before IAC was formally launched by the MOH. During this period, IDI was still piloting the IAC strategy in some health facilities. Relatedly a study done in Zimbabwe found a 75.7% coverage of IAC. This was also done in the first year of the Zimbabwe National ART program adopting the 2016 WHO guidelines on IAC strategy [15]. During this short implementation period, HCWs might not adequately have understood IAC procedures.

Besides improving VL suppression in 44.1% of the targeted population, IAC had a significant relative effect of 22% in achieving suppression compared to the counseling strategies that were used before it was introduced. To our knowledge, this is the first study in Uganda and the region to assess the relative effect of IAC in routine HIV care program. The improvement of VL suppression in our study is similar to Bonner et al. findings which showed that patients with unsuppressed VL always suppress following an adherence intervention [21]. However, for the current study, IAC did not produce the expected proportions of VL suppression (above 70%) as reported in Bonner et al. [21]. Our findings are similar to Nassuna et al. and Bvochora et al.’s findings which also produced lower proportions of VL suppression which were 23% [42] and 31.2% [15] respectively. Our findings show that, IAC sub-optimally improved VL suppression at KHCIV despite wide coverage. The effectiveness of IAC could have been reduced by the prevailing health system and patient related challenges. There is a need to further assess the quality and fidelity of IAC counseling sessions and procedures to further understand why IAC was sub-optimally effective in such settings.

In this study PI-based ART regimens were associated with reduced VL suppression compared to other regimens despite requiring less adherence percentage levels (80% versus 95%) [8, 9, 43]. This may probably be due to the dosing demands of the PI-based regimens visa-vis the reason for poor adherence. It is possible that the twice dosing demand of the regimen caused non-adherence and hence failure to achieve viral suppression. This may mimic the reverse causality concept as it has been reported in other studies [44–46]. For instance, HCW’s interviews revealed that hesitance to take drugs on empty stomach due to lack of food and non-disclosure especially to sexual partners that results in taking drugs while hiding are among the major reasons for unsuppressed VL during IAC; these are likely to worsen more for a twice dosing regimen than a single dose regimen. Additionally, PI-based side effects such as diarrhea may cause poor adherence and hence unsuppressed VL. Another reason for unsuppressed VL in our study might be virological failure which was reported to be high among patients on PI-based regimen in a study done in Tanzania [47]. Relatedly, Deborah et al. found higher odds of unsuppressed VL among adolescents aged 18–20 years on PI based regimen compared to those on single dose regimens, although their results were not statistically significant [48]. Our findings are contrary to Bvochora et al.’s findings in Zimbabwe where patients on 2nd line ART (PI-based) were 65% more likely to re-suppress compared to those on 1st line regimen following adherence counseling [15]. Similarly, other studies in South Africa also showed that viremic patients on second-line ART usually achieve a suppressed VL after targeted IAC [49, 50]. Jobanputra et al. did not find a statistical difference in achieving VL suppression between patients on 1st and 2nd regimens [22]. However, all these studies were non-comparative unlike the current study. Therefore, our results imply that HCW should take caution and be more vigilant while offering IAC to unsuppressed PLHIV on PI-based regimens.

Patient related factors including non-disclosure, social economic constraints, relocations and gender-based violence; and health care system related factors including lack of privacy at the facility, lack of skills among HCW and lack of a multidisciplinary team negatively affected the program implementation and the likely VL suppression during IAC. These findings are similar to previous findings where it was reported that non-disclosure was a major barrier in achieving ART adherence and eventually VL suppression [51–54], social economic constraints may act as a barrier for transportation to the facility for drug refills or cause the patient to fear taking medication on empty stomach due to lack of food [29, 55–57], relocations may lead to lost to follow up [58, 59] and gender based violence [54, 60–63] may lead to psychological stress to a patient. Additionally, Health care system related barriers to ART adherence have been reported especially in resource limited settings [64]. For example Odokonyero et al. reported that healthcare infrastructure and health care work force are vital in effectiveness of a health intervention or strategy [27]. Health care system related factors such as lack of client privacy during HIV care and lack of skills among HCW [28, 29, 54, 65], poor quality of counseling sessions [26] and shortage of HCW or cadres and lack of multidisciplinary team [66–68] hinder provision of an effective adherence intervention.

The effectiveness of IAC was assessed up to 31st October 2018 before the Covid-19 outbreak. However, in the interviews with HCW, which were conducted during the Covid-19 pandemic revealed that the pandemic further jeopardized ART adherence and provision of IAC. The impact of the Covid-19 pandemic on service delivery and uptake has been reported in other studies [69–71]. A part from draining the available limited resources toward management of Covid-19 patients, HIV clients could not easily access care and drug refills due to restrictions on movements and transport challenges. This led to interruption of IAC appointments as well as ART supply. Moreover, interruption of ART supply is the most important determinant of HIV-related mortality [69].

Provision of IAC may be facilitated by use of peer educators and assigning special clinic days to particular client groups. Peer educators are instrumental in provision of HIV services elsewhere [72–74]. Peer based counseling is effective in provision of health services [75–77]. However, peers should have adequate training and support to ensure quality services [78, 79]. Assigning a special clinic day for a group of clients with similar adherence issues improves efficiency and effectiveness in the provision of a service. During these special clinic days, individual or group counseling strategy may be provided. For example, children with adherence difficulties are given a special day together with their parents or guardians. This allows extensive group interaction and discussion between HCW and clients on adherence issues that may be affecting these children. The group counseling strategy has also been used elsewhere [80].

### Strengths and limitations

This is one of the few studies to assess the effect of an adherence intervention using a comparison group. The use of routine program data with limited variables and data quality issues may limit the analysis and conclusions. The study used “as treated” analysis strategy which may have biased results in favor of the IAC intervention period since 76 participants in the pre-intervention period (Jan 2015–Dec2016) received IAC. However, this may have had minimal effect due to the fact that the study aimed at the effect of the intervention not the effect of the randomization or grouping. The study excluded the clients who were no longer active in the clinic which would favor the effectiveness of IAC if most of those who were no longer active in the clinic had a non-favorable outcome of unsuppressed VL following IAC. However, non-active clients were not considered in both groups and thus balances this effect across the two groups.

## Conclusions and recommendations

At KHCIV, IAC was fully embraced, was optimally provided to the target population and was more effective in VL suppression than regular routine counseling. However, its full potential was not reached due to a combination of patient and health care system related factors. Clients on PI-based regimen were less likely to suppress during IAC probably due to dosing demands of the regimen but peer educators and having special clinic days for particular clients were important during provision of IAC. The findings highlight a need; to avail adequate requirements for conducting IAC as stipulated in IAC program manuals and guidelines including a multidisciplinary team, HCW and privacy; to provide client or patient centered approaches while handling specific client adherence difficulties such as drug specific challenges, non-disclosure, financial constraints and gender-based violence etcetera; and health facilities with limited workforce should utilize peer educators and special clinic days during provision of IAC.

## Data Availability

The datasets used and/or analyzed during the current study are available from the corresponding author on reasonable request.

## Abbreviations

AIDs: Acquired Immunodeficiency syndrome
ART: Anti-Retroviral Therapy
GBV: Gender-Based Violence
HDREC: Makerere University School of Public Health Higher Degrees, Research and Ethics Committee
HCWs: Health care worker
HIV: Human Immunodeficiency Virus
IAC: Intensive Adherence Counseling
IDI: Infectious Disease Institute
KCCA: Kampala Capital City Authority
KHCIV: Kisenyi Health Center IV
OVC: Orphans and vulnerable children
PLHIV: People living with HIV
PI: Protease Inhibitor
PR: Prevalence Ratios
SSA: Sub-Saharan Africa
VL: Viral Load
WHO: World Health Organization
